# Retroperitoneal hemorrhagic shock in a patient on warfarin therapy

**DOI:** 10.4103/0974-2700.50752

**Published:** 2009

**Authors:** Sankar Subramanian, Subramanian Marappa Gounder, Arunkumar Thirunarayanan, Anand Kannan, Nandigam Venu

**Affiliations:** Department of Surgical Gastroenterology, Sri Ramachandra Medical College & Research Institute, Sri Ramachandra University, Porur, Chennai, Tamilnadu, India

**Keywords:** Warfarin, hemoperitoneum, shock

## Abstract

Oral anticoagulants are an established treatment modality in the prophylaxis of thromboembolic events in various clinical scenarios. Needless to say that, bleeding is a natural adverse effect of this drug. Most of the times bleeding is inconsequential. But nevertheless massive and fatal bleeding can occur occasionally. The case reported here is rare, as the patient presented with massive hemoperitoneum due to mesenteric hemorrhage and hemorrhagic infarction of small bowel necessitating Laparotomy resection.

## INTRODUCTION

Bleeding is an adverse effect of warfarin therapy and generally such episodes are inconsequential. It is rare to have patients present with massive and near fatal bleeding. Our patient is one such case.

## CASE REPORT

**Figure 1:**
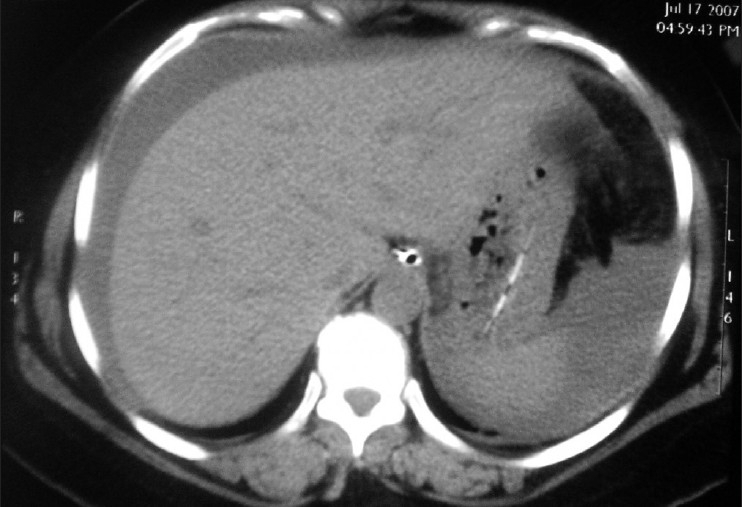
Sub-diaphragmatic fluid collection with a CT attenuation value of 30 suggesting hemoperitoneum

**Figure 2:**
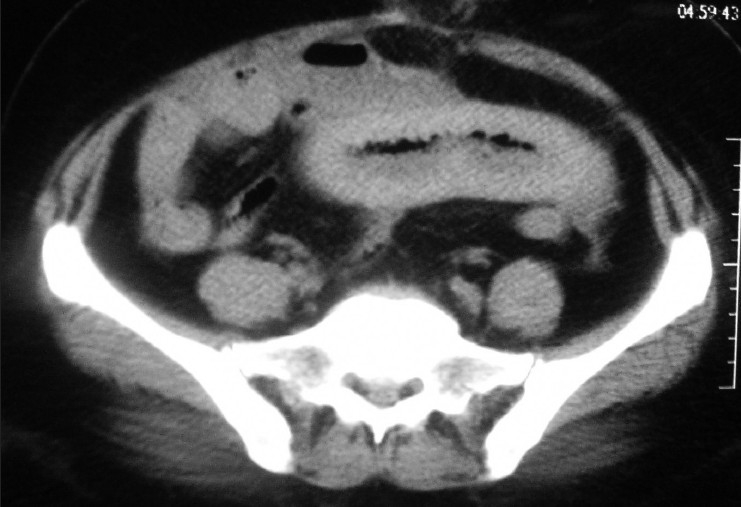
A segment of small intestine is grossly thickened

**Figure 3:**
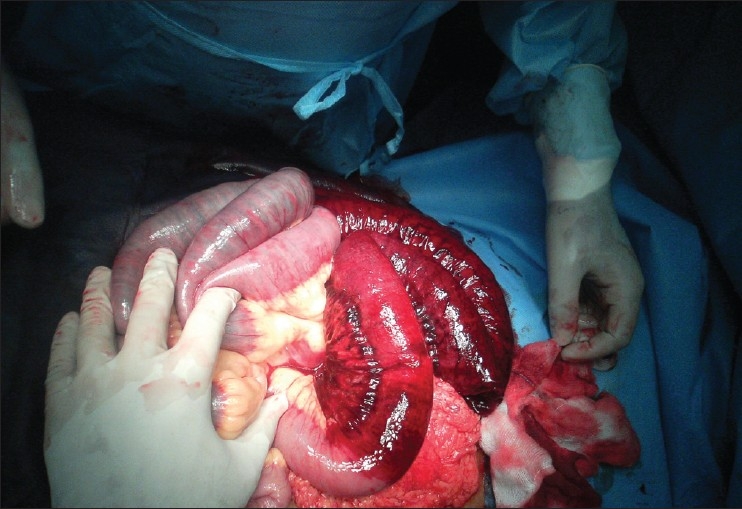
Segment of small intestine is severely congested due to mesenteric hemorrhage

We encountered a 43-year-old female patient who presented in shock with cool clammy extremities, tachycardia with a rate of 135 and blood pressure of 80/50 mm Hg. This was preceded by a history of 12h of abdominal pain and progressive distention without a history of trauma. The patient was alert oriented to time place and person with a oxygen saturation of 94% and respiratory rate of 24. Her medical history was positive for a stroke 1 year ago for which she was on warfarin and clopidogrel. We could not find out whether she was allergic to aspirin. We started the primary treatment of shock and bolused her with intravenous fluids, started nasal oxygen, put her on a monitor and collected cultures and blood for complete blood count, differential, electrolytes, type and cross, renal and liver profile. An International Normalize Ratio (INR) test and Prothrombin time were also ordered. Suspecting the worse case scenario of internal hemorrhage secondary to warfarin toxicity we performed a bedside ultrasound which was positive for free fluid in the abdomen. Her laboratory values came back with hemoglobin of 4 gm%, a normal white count, platelet count and an INR of 8 which was way beyond the normal range. After stabilizing her with intravenous normal saline boluses of 2 L, we did a computerized tomography (CT) scan of the abdomen and pelvis to identify a visceral source of bleeding. CT abdomen showed free fluid in the sub diaphragmatic spaces with CT attenuation value of 30 suggesting hemoperitoneum [Figure 1]. CT also showed a segment of grossly thickened bowel loop [Figure 2]. The patient was taken to the operating room for an exploratory laparotomy. More than 2 L of blood was suctioned from the abdomen. There were 2 ft of small bowel and its mesentery which were found to be hemorrhagic and severely congested [Figure 3]. No active sites of bleeding could be identified. The source of bleeding was probably mesenteric vessels. Since the segment of bowel did not appear healthy it was resected and end-to-end anastomosis was performed. Patient had an uneventful postoperative recovery. Anticoagulants were withdrawn.

## DISCUSSION

Warfarin is an oral anticoagulant most frequently used to control and prevent thromboembolic disorders. Prescribing the dose that both avoids hemorrhagic complications and achieves sufficient suppression of thrombosis requires a thorough understanding of the drug's unique pharmacology. Warfarin has a complex dose-response relationship that makes safe and effective use a challenge. Warfarin exerts its action by inhibiting Vitamin K dependent coagulation factors (II, VII, IX and X). It also inhibits the synthesis of natural anticoagulants in the blood, protein C and S. Due to the difference in the half life of “coagulation factors” and “anticoagulants”, the coagulation system may be transiently biased towards clotting after starting warfarin. Hence one should start warfarin only after anticoagulating the patient with heparin, lest one may end up in thrombosis. The target International normalized ratio (INR) is maintained around 2–3. Spontaneous bleeding is one of the well reported and most common adverse effects of warfarin.[1] Bleeding is usually subcutaneous or intramuscular. Management is generally to reduce the dose of warfarin. Intracranial, retroperitoneal and gastrointestinal bleeding can be life threatening. Gastrointestinal bleeding is usually intramural which can be treated conservatively with bowel rest and adjusting the dose of warfarin.[2] The unmasking of previously unidentified bowel tumors has been described after spontaneous warfarin-associated bleeding.[3] Spontaneous mesenteric hemorrhage with hemorrhagic infarction of small intestine is extremely rare.[4] Various factors determine the bleeding complications of Warfarin. These include older age,[5] dose, duration of therapy, drug interaction and occult diseases. Many drugs interact with warfarin, the common being NSAID. In fact the most common cause of bleeding secondary to warfarin is drug interaction.[6] It has also been shown that warfarin when combined with antiplatelet drugs like clopidogrel have higher incidence of bleeding.[6] There are reports which suggest that warfarin-induced bleeding is on the rise.[6] Dedicated monitoring of the coagulation profile in patients taking long-term warfarin is mandatory to prevent this complication.

## CONCLUSION

Long term Warfarin therapy needs close monitoring with repeated measurements of the INR. Physicians and patients have to be aware that there is increased risk of bleeding when on warfarin therapy. Our patient had a very high INR indicative of warfarin toxicity which was an important cause for the bleeding in her abdomen
